# Bacterial communities in different sections of a municipal wastewater treatment plant revealed by 16S rDNA 454 pyrosequencing

**DOI:** 10.1007/s00253-012-4082-4

**Published:** 2012-05-05

**Authors:** Lin Ye, Tong Zhang

**Affiliations:** Environmental Biotechnology Lab, Department of Civil Engineering, The University of Hong Kong, Pokfulam Road, Hong Kong SAR, China

**Keywords:** WWTP influent, WWTP effluent, Activated sludge, Digestion sludge, 454 Pyrosequencing, Bacterial community

## Abstract

In this study, we successfully demonstrated that 454 pyrosequencing was a powerful approach for investigating the bacterial communities in the activated sludge, digestion sludge, influent, and effluent samples of a full scale wastewater treatment plant treating saline sewage. For each sample, 18,808 effective sequences were selected and utilized to do the bacterial diversity and abundance analysis. In total, 2,455, 794, 1,667, and 1,932 operational taxonomic units were obtained at 3 % distance cutoff in the activated sludge, digestion sludge, influent, and effluent samples, respectively. The corresponding most dominant classes in the four samples are *Alphaproteobacteria*, *Thermotogae*, *Deltaproteobacteria*, and *Gammaproteobacteria*. About 67 % sequences in the digestion sludge sample were found to be affiliated with the *Thermotogales* order. Also, these sequences were assigned into a recently proposed genus *Kosmotoga* by the Ribosomal Database Project classifier*.* In the effluent sample, we found high abundance of *Mycobacterium* and *Vibrio*, which are genera containing pathogenic bacteria. Moreover, in this study, we proposed a method to differentiate the “gene percentage” and “cell percentage” by using Ribosomal RNA Operon Copy Number Database.

**Electronic supplementary material:**

The online version of this article (doi:10.1007/s00253-012-4082-4) contains supplementary material, which is available to authorized users.

## Introduction

Biological treatment processes are the most widely used approach for treating municipal and industrial wastewater in wastewater treatment plants (WWTPs) due to their high efficiency for various organic/nutrient matters removal and low operational cost. The microbial community, which is dominated by bacteria (Wagner et al. 2002), plays an essential role in the biological treatment reactors and has been studied for several decades by both isolation (Neilson 1978) and molecular methods, such as polymerase chain reaction (PCR)-denaturing gradient gel electrophoresis (Muyzer et al. 1993; Ye and Zhang 2010), terminal restriction fragment length polymorphism (Liu et al. 1997), cloning (Schuppler et al. 1995), and fluorescent in situ hybridization (Erhart et al. 1997). The culturing methods have been a very direct and effective way to characterize the microbial community. However, most of the bacteria in the natural environment cannot be cultured in an artificial medium in the laboratory (Giovannoni et al. 1990; Hugenholtz et al. 1998) and the diversity of the uncultured bacteria is quite considerable (Whitman et al. 1998). The molecular methods greatly promoted our understanding of the microbial community. But for complex environmental samples (such as soil and activated sludge) with overwhelming genetic diversities, these methods are still far away from revealing the panorama of the bacterial community and can only investigate the most abundant population in these samples (Claesson et al. 2009).

The next generation sequencing technologies originated several years ago made the high throughput sequencing easy to be implemented with low cost (Glenn 2011). 454 Pyrosequencing is one of the popular high throughput sequencing systems, which can generate more than 400,000 effective reads with average read length up to several hundred base pairs and average quality of greater than 99.5 % accuracy rate (Droege and Hill 2008; Glenn 2011). By incorporating barcode sequences on primers, a certain number of DNA samples can be sequenced at the same time in one run. This technology has been successfully used in investigating microbial diversity and abundance in various samples, such as marine water (Qian et al. 2010), soil (Roesch et al. 2007), and human distal intestine (Claesson et al. 2009). There are also several studies applying pyrosequencing in exploring the microbiota in WWTP (Kim et al. 2011; McLellan et al. 2010; Ye et al. 2011); however, these results are very limited and preliminary.

In this study, to investigate the bacterial diversity and abundance in a full scale WWTP treating saline sewage in Hong Kong, we systematically analyzed 16S rRNA gene in the influent, activated sludge, digestion sludge, and effluent by using 454 high throughput pyrosequencing. It was found that the bacterial diversities in the four samples are quite different. Besides, in this study, we also introduced a method to consider and compare the difference of 16S rRNA gene percentage and bacteria cell number percentage in these samples. The results showed that some genera, especially the abundant genera, may be underestimated or overestimated when using 16S rRNA gene to reflect their abundances.

## Materials and methods

### WWTP description and sampling

In this study, the activated sludge, digestion sludge, influent, and effluent samples were taken from Shatin WWTP, which is a full scale municipal wastewater treatment plant in Hong Kong. Because seawater has been used extensively in the toilet flushing system in Hong Kong, this WWTP treats saline sewage (salinity ~1.2 %) containing about 30 % seawater with activated sludge system. It treats about 216,000 m^3^ wastewater per day with a COD concentration of 226 ~ 491 mg l^−1^. The aeration tank was partitioned into two zones (anoxic zone and aerobic zone) for carbon and nitrogen removal but no special facilities for phosphate removal. The activated sludge sample analyzed in this study was taken from the aerobic zone. To reduce sludge volume, both the primary sludge and the surplus activated sludge are digested in mesophilic anaerobic digesters. Detailed information about the WWTP and the samples were summarized in Table S4 and Fig. S3. When taking samples, the activated sludge (mixed liquid containing both flocs and suspended bacteria in the aerobic zone of the aeration tank) and the digestion sludge were taken from the reactor and mixed thoroughly and then fixed on site by mixing with 100 % ethanol at a volume ratio of 1:1 and kept in an ice box for transportation and then stored in our laboratory at −20 °C before DNA extraction. Influent and effluent samples were kept in plastic containers and delivered to our laboratory within 3 h. Immediately after arriving at the laboratory, 12 ml influent was centrifuged at 4,000 rpm for 10 min at 4 °C and 400 ml effluent was filtrated using a 0.45-μm glass fiber filter to collect the bacteria cells. The collected residue was used for DNA extraction.

### DNA extraction and PCR

For all samples in this study, DNA was extracted using FastDNA® SPIN Kit for Soil (MP Biomedicals, Illkirch, France). Before pyrosequencing, the above DNA of each sample was amplified with a set of primers targeting the hypervariable V4 region of the 16S rRNA gene (RDP’s Pyrosequencing Pipeline: http://pyro.cme.msu.edu/pyro/help.jsp). The forward primer is 5′-AYTGGGYDTAAAGNG-3′ and the reverse primers are the mixture of four primers, i.e., 5′-TACCRGGGTHTCTAATCC-3′, 5′-TACCAGAGTATCTAATTC-3′, 5′-CTACDSRGGTMTCTAATC-3′, and 5′-TACNVGGGTATCTAATCC-3′ (Claesson et al. 2009). Barcodes that allow sample multiplexing during pyrosequencing were incorporated between the 454 adaptor and the forward primers. The PCR amplification was conducted in a 100-μl reaction system using MightyAmp polymerase (TaKaRa, Dalian, China). The amplification was conducted in an i-Cycler (BioRad, Hercules, CA, USA) under the following conditions: 98 °C for 2 min, 28 cycles at 98 °C for 15 s, 56 °C for 20 s and 68 °C for 30 s, and a final extension at 68 °C for 10 min. The PCR products were purified by using PCRquick-spin^TM^ PCR Product Purification Kit (iNtRON Biotechnology, South Korea) and then mixed equally before conducting pyrosequencing.

For clone library construction, the DNA of another digestion sludge sample taken from the sample anaerobic digester was amplified by PCR using universal bacterial 16S rRNA primer set EUB8F (5′-AGAGTTTGATCMTGGCTCAG-3′) (Heuer et al. 1997) and UNIV1392R (5′-ACGGGCGGTGTGTRC-3′) (Ferris et al. 1996). The vector used for ligation was pMD^®^18-T (TaKaRa). The purified plasmid was sequenced on an ABI 3730xl capillary sequencer (Applied Biosystems, Foster City, CA, USA).

### High throughput pyrosequencing

The PCR products of the V4 region of 16S rRNA gene were sequenced using the Roche 454 FLX Titanium sequencer (Roche, Nutley, NJ, USA). Samples in this study were individually barcoded to enable multiplex sequencing. The results are deposited into the NCBI short reads archive database (accession number: SRA026842.2).

### Sequence processing and bacterial population analysis

Following pyrosequencing, Python scripts were written to (1) remove sequences containing more than one ambiguous base (‘N’), (2) check the completeness of the barcodes and the adaptor, and (3) remove sequences shorter than 150 bp.

454 Sequencing noises were removed by Pre.cluster (Huse et al. 2010) tool in Mothur package (Roeselers et al. 2011). Chimeras introduced in the PCR process were detected using ChimeraSlayer (Haas et al. 2011) in Mothur package. Because all sequences flagged as chimeras are not recommended to be discarded blindly (http://microbiomeutil.sourceforge.net/), so the reads flagged as chimeras were submitted to Ribosomal Database Project (RDP) classifier (Wang et al. 2007; Cole et al. 2009). Those being assigned to any known genus with 50 % confidence threshold were merged with the non-chimera reads to form the collection of “effective sequences” for each sample.

**Fig. 1:**
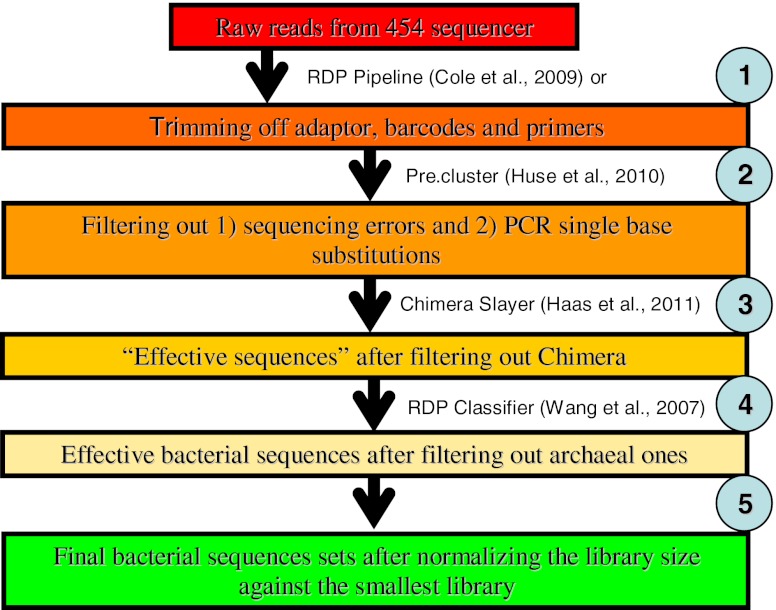
Sequences quality trimming flow chart

Although the primers used in this study are bacteria-specific primers, a few archaeal sequences might be obtained. The same situation was also observed in another study (Qian et al. 2010). To remove these archaeal sequences, the effective sequences of each sample were submitted to the RDP classifier again to identify the archaeal and bacterial sequences, and the archaeal sequences were filtered out using a self-written Python script. The average length of all effective bacterial sequences without the primers was 207 bp. The above quality trimming process was summarized in Fig. 1.

After that, the “RDP Align” tool in RDP’s Pyrosequencing Pipeline was used to align the effective bacterial sequences of each sample. A cluster file was generated for each sample with “RDP Complete Linkage Clustering” tool. With the cluster file, the rarefaction curves were generated using the “RDP Rarefaction” tool.

All effective bacterial sequences obtained from pyrosequencing in this study were compared with Greengenes 16S rRNA gene database (DeSantis et al. 2006) annotated with NCBI taxonomy using NCBI’s BLASTN tool (Altschul et al. 1990) and the default parameters except for the maximum hit number of 100 (Claesson et al. 2009). Then, the sequences were assigned to NCBI taxonomies with MEGAN (Huson et al. 2007) by using the lowest common ancestor algorithm and the default parameters except the BLAST bitscore, for which we found three values used in previous studies: 35 (Huson et al. 2007), 86 (Urich et al. 2008), and 250 (Claesson et al. 2009). In this study, the intermediate BLAST bitscore cutoff threshold 86 was applied.

After the sequences were assigned to NCBI taxonomies, the percentage of bacteria in each taxon could be calculated, which was defined as “gene percentage” in this study. From the Ribosomal RNA Operon Copy Number Database (rrnDB) (http://rrndb.mmg.msu.edu), we downloaded the data of rRNA operon copy numbers of each genus. According to these copy number data, the abovementioned “gene percentage” could be converted to “cell percentage”. It was noted that a few genera found in this study have no rRNA operon copy numbers in rrnDB. For those genera, we used the average rRNA operon copy number values in the whole rrnDB. And if the species in a genus contains different rRNA operon copy numbers, we used the average value of all the species in this genus.

## Results

### Effectiveness check for the raw reads

In this study, we obtained 51,072, 47,072, 45,577, and 46,785 reads for the activated sludge, digestion sludge, influent, and effluent samples, respectively. After initial quality check mentioned in the “Materials and methods” section, the chimera and *Achaea* sequences were also checked and filtered. As shown in Table 1, 43–65 % of the raw reads met the quality and length criteria. By using ChimeraSlayer in Mothur package, 10–20 % of the reads were flagged as chimeras in the activated sludge and influent and effluent samples, while only 2.6 % reads in the digestion sludge sample were judged to be chimeras. Some of these so-called chimeras may represent naturally formed sequences that do not represent PCR artifacts and are not recommended to be blindly discarded (http://microbiomeutil.sourceforge.net/). So, in this study, all the chimeras picked by ChimeraSlayer were submitted to RDP classifier for further checking and it was found that about half (shown in Table 1) of the chimeras can be assigned to a known genus at 50 % confidence threshold. Hence, only those sequences that cannot be assigned into a genus were regarded as real chimeras and excluded in the downstream analysis. Although the primers used in this study were specific for bacteria, some sequences were assigned to *Archaea* by the RDP classifier. These sequences were filtered out in this step. At last, in order to do the comparison at the same sequencing depth, 18,808 effective bacterial sequences were extracted from each sample to do the downstream analysis.

**Table 1:** Sequences from the four samples

Sample name	Reads		Chimeras		Sequences				3 % distance				6 % distance			
	Raw read	Effective	Assigned to genus	Not assigned to genus	Effective	Archaeal	Bacterial	Normalization	Chao 1 richness estimation	Shannon diversity	OTU	Good’s coverage (%)	Chao 1 richness estimation	Shannon diversity	OTU	Good’s coverage (%)
Activated sludge	51,072	28,266	2,565	3,289	24,977	16	24,961	18,808	3,902	6.42	2,455	94.2	2,700	6.12	1,852	96.2
Digestion sludge	47,072	30,488	251	533	29,955	357	29,598	18,808	1,618	2.35	794	97.7	1,140	2.12	630	98.3
Influent	45,577	21,861	1,172	1,192	20,669	42	20,627	18,808	2,868	5.26	1,667	95.8	1,815	4.91	1,172	97.4
Effluent	46,785	20,250	1,969	1,421	18,829	21	18,808	18,808	3,711	5.54	1,932	94.9	2,671	5.25	1,562	96.3

### Bacterial community composition

**Fig. 2:**
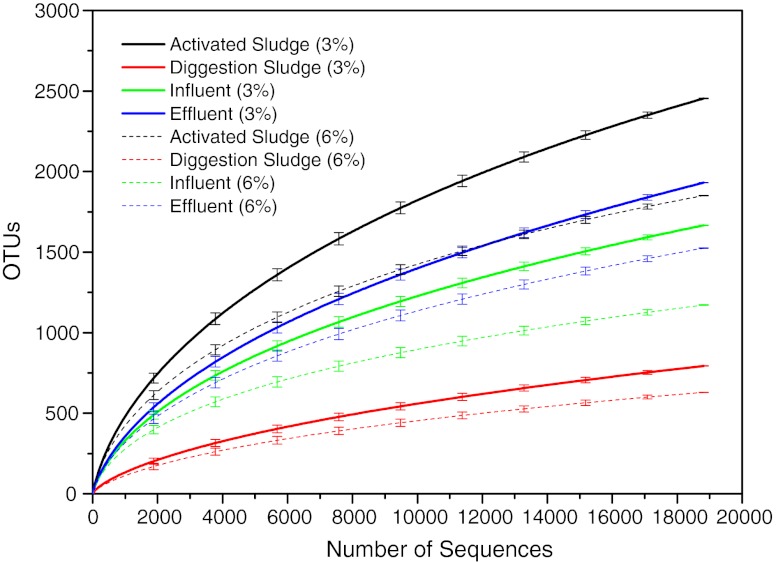
Rarefaction curves of the four samples at cutoff levels of 3 % (*solid lines*) and 6 % (*dash lines*) created by using RDP’s pyrosequencing pipeline. The *error bars* show 95 % confidence of upper and lower limits

In order to compare the bacterial species richness among these samples, operational taxonomic units (OTUs) were determined for each sample at distance levels of 3 and 6 % (Table 1). The OTU number in the activated sludge was the largest among the four samples, i.e., 2,455 and 1,852 at distance cutoff levels of 3 and 6 %, respectively. And the digestion sludge contained the least OTU amount. The bacterial phylotype richness levels can also be reflected using Shannon diversity index (Table 1) which also revealed that the activated sludge had the highest bacterial diversity among the four samples. The rarefaction curves of the four samples at distance cutoff levels of 3 and 6 %, as shown in Fig. 2, demonstrated clearly that the bacterial phylotype richness of the activated sludge was much higher than the other samples. The influent and effluent samples had moderate richness and the digestion sludge had the least richness. The rarefaction curves, especially those of the activated sludge, influent, and effluent samples, did not level off even at the sequencing depth of 18,808, suggesting that this sequencing depth was still not enough to cover the whole bacterial diversity and thus further sequencing would be valuable to detect more species.

**Fig. 3:**
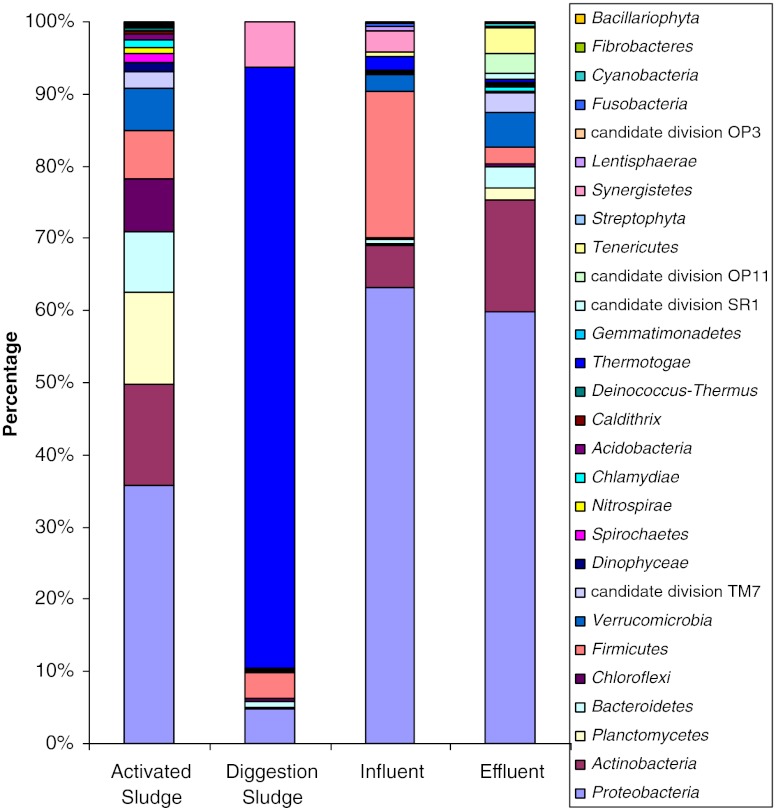
Relative abundances of different phyla in the four samples (the results were obtained by using BLASTN combined with MEGAN)

The effective bacterial sequences in the four samples were all assigned to corresponding taxonomies by using BLAST combined with MEGAN. The relative abundances of different phyla in the four samples were shown in Fig. 3. From the phylum assignment result, it was found that the bacterial diversity in the digestion sludge was significantly different from the other three samples. Over 80 % of the sequences in the digestion sludge were assigned into *Thermotogae*, which is a phylum that may have much in common with species in *Archaea* (Cracraft and Donoghue 2004). In other samples, *Proteobacteria* were the dominant phylum, accounting for about 36, 63, and 60 % in the activated sludge, influent, and effluent samples, respectively. *Actinobacteria* were the secondary phylum in the activated sludge and effluent samples, corresponding to the percentages of about 14 and 15 %. However, in the influent sample, the secondary phylum was *Firmicutes* at a percentage of 20.26 %. The total phyla number in the activated sludge, digestion sludge, influent, and effluent samples were 23, 13, 19, and 23, respectively, suggesting that the bacterial diversity in the digestion sludge was lower than the other samples even at the phylum level. There were in total 12 phyla shared by the four samples, which were *Proteobacteria*, *Actinobacteria*, *Planctomycetes*, *Bacteroidetes*, *Chloroflexi*, *Firmicutes*, *Verrucomicrobia*, *candidate division TM7*, *Thermotogae*, and *Synergistetes.* The detailed comparison of these phyla in the samples was shown in Table S1.

**Fig. 4:**
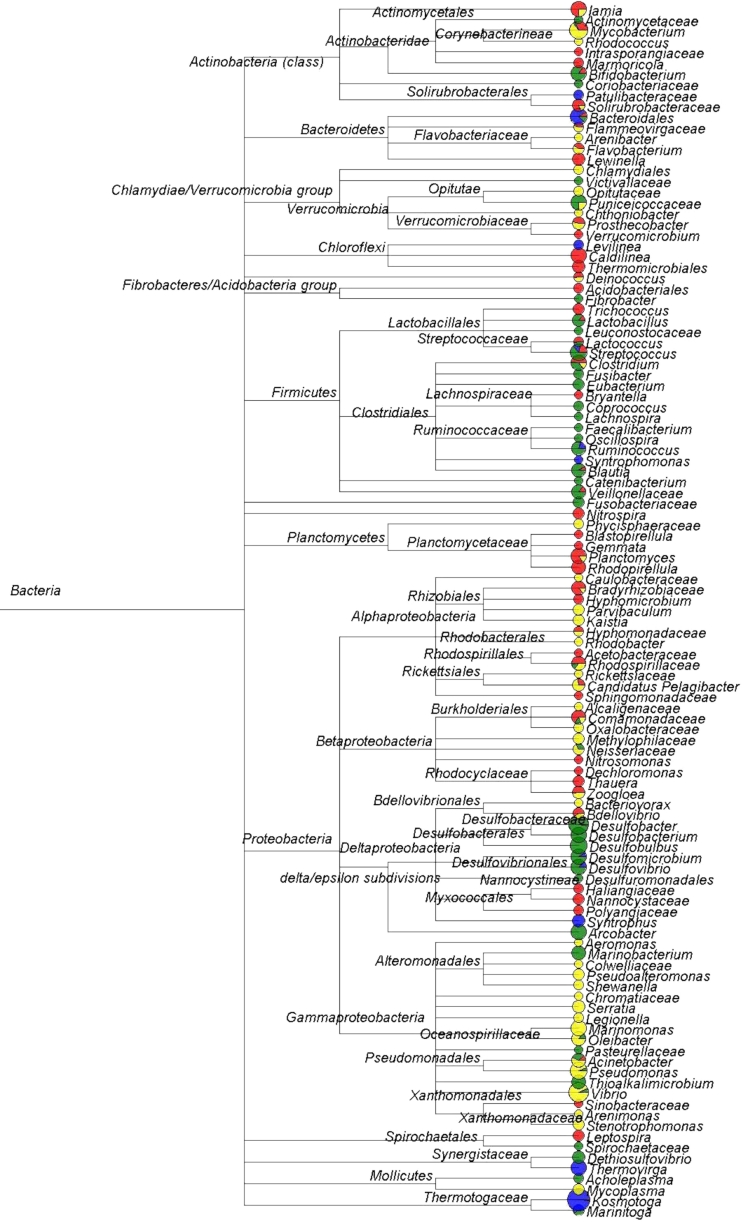
Sequences assignment results at the genus level. All effective sequences in the four samples were assigned into NCBI taxonomies by using BLASTN and MEGAN. Only nodes with over 30 sequences were shown in this figure. *Pie charts* indicate the relative sequence abundances of the corresponding nodes in the four samples. *Red* activated sludge, *blue* digestion sludge, *green* influent, *yellow* effluent

Besides the phylum, bacterial diversity and abundance were also analyzed more specifically at other taxonomic units, i.e., class (Table S2, order (Table S3), and genus (Fig. 4). In any of the taxonomic units, the bacterial diversity of the digestion sludge was always found dramatically different from those of other samples. Only a few sequences assigned into the taxonomic units shared with the other samples. It is interesting to find that the most dominant classes in the activated sludge, digestion sludge, influent, and effluent are different, which were *Alphaproteobacteria*, *Thermotogae*, *Deltaproteobacteria*, and *Gammaproteobacteria*, respectively. At the order level, it was found that the top five dominant populations in the activated sludge samples were *Planctomycetales*, *Actinomycetales*, *Rhizobiales*, *Caldilineales*, and *Sphingobacteriales*, which were totally different from those dominant populations of the influent samples, i.e., *Desulfobacterales*, *Clostridiales*, *Desulfovibrionales*, *Lactobacillales*, and *Bifidobacteriales*. This probably indicated that some bacterial populations in the influent may not proliferate in the activated sludge. Figure 4 shows the comparison of the sequence assignment results on the genus level. It was found that at the genus level of MEGAN’s cladogram, there were 11 nodes shared by the activated sludge and influent sample and 17 nodes shared by the activated sludge and the effluent samples. However, there were only five nodes shared by the activated sludge, influent, and effluent samples.

### High abundance of *Thermotogales* in the digestion sludge

In this study, we observed extremely high abundance of *Thermotogales* that exists in the anaerobic digestion samples. According to the taxonomic analysis results of BLAST combined with MEGAN, there were 12,594 out of the totally 18,808 sequences assigned to the *Thermotogales* order, corresponding to a percentage of 67 % (Table S3). Using the RDP classifier, these high abundant sequences were further assigned into the *Thermotogaceae* family and *Kosmotoga* genus (Fig. S1) at a confidence threshold of 50 %. The high abundance of *Kosmotoga* in this digestion sludge was further confirmed by another independent sample from the same digester using the cloning library method with the universal bacterial 16S rRNA gene primers (EUB8F and UNIV1392R). Among 60 clones sequenced by the Sanger method, 24 of them were assigned to *Thermotogales* order and *Kosmotoga* genus by the RDP classifier (Fig. S2).

### Differences between “gene percentage” and “cell percentage”

**Fig. 5:**
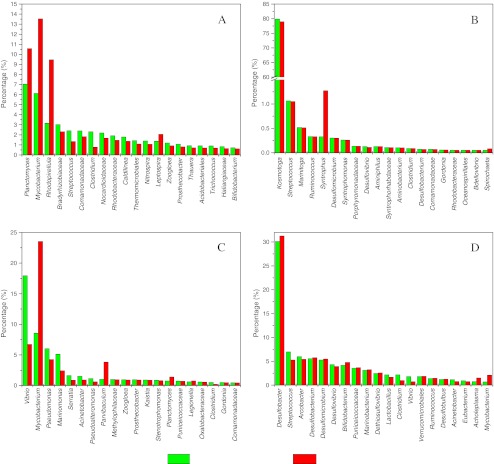
Top 20 genera in each sample showing the comparison of percentages of 16S rRNA gene number and cell number (**a** activated sludge, **b** digestion sludge, **c** effluent, **d** influent)

In previous studies, 16S rRNA gene percentage was often used to reflect the bacterial abundance in the samples (Bibby et al. 2010; Park et al. 2008; Ye et al. 2011). However, the copy numbers of 16S rRNA gene in prokaryotic microorganisms are not equal and may vary from 1 to 15 (Klappenbach et al. 2001). So, the cell percentages may disagree with the 16S rRNA gene percentages. In this study, we converted the 16S rRNA gene numbers into cell number at the genus level referring to the rrnDB and linked the two percentages commonly used in environmental microbiology (Lee et al. 2009). Figure 5 shows the comparison of the percentage of 16S rRNA gene and cells of the four samples, i.e., activated sludge, digestion sludge, influent, and effluent. The result indicated that there were a few genera that could be significantly overestimated or underestimated. The 16S rRNA gene percentages of the three genera (*Planctomyces*, *Mycobacterium*, and *Rhodopirellula*) in the activated sludge sample, two genera (*Mycobacterium* and *Parvibaculum*) in the effluent sample, and one genus (*Syntrophus*) in the digestion sludge were lower than their corresponding cell percentage. Moreover, one genus (*Vibrio*) in the effluent sample had a higher 16S rRNA gene copy percentage than its cell percentage. In the influent sample, the percentage of 16S rRNA gene copy and the cell number matched with each other consistently for most of the genera. The largest difference observed in this study was the *Syntrophus* genus in the digestion sludge sample, which was up to 389 %.

## Discussion

In this study, we analyzed the bacterial diversity in a municipal WWTP. The results show that activated sludge harbored the highest diversity of bacteria. At a distance cutoff of 3 %, as shown in Table 1, 2,455 OTUs were obtained from 18,808 sequences, indicating that activated sludge is a highly species-rich ecosystem, which is more complicated than seawater (Qian et al. 2010) and comparable to soil (Roesch et al. 2007). The influent and effluent samples contained moderate amounts of bacterial populations. The OTU number of the influent in the present study was much smaller than a previous study conducted on a WWTP influent (McLellan et al. 2010), in which over 3,000 OTUs were identified with sequencing depths between 17,338 and 34,080 V6 region reads for each sample. The digestion sludge contained the least OTUs and most of the sequences (67 %) concentrated in the order *Thermotogales*.

Regarding the bacterial community in the activated sludge, so far, only very few studies have been conducted, especially with the activated sludge in the full scale municipal WWTPs. Snaidr et al. investigated the bacterial community structure of activated sludge of a large municipal WWTP by using cloning methods (Snaidr et al. 1997). In those days, due to the lack of a high throughput approach, their results were very preliminary. *Proteobacteria* was found to be the dominant phylum, which was consistent in this study. Their cloning result showed that *Betaproteobacteria* was the most abundant class in the phylum of *Proteobacteria*, followed by *Gammaproteobacteria*. However, the results in the present study suggested that *Alphaproteobacteria* and *Actinobacteria* were the top two classes in the activated sample (Table S2). Another recently published study analyzed the bacterial diversity of a full-scale integrated fixed-film activated sludge system using the high throughput pyrosequencing method (Kwon et al. 2010). Their results also showed that *Betaproteobacteria* and *Gammaproteobacteria* were the most abundant groups in the activated sludge. The inconsistence between the present study and other studies may be caused by the different conditions, especially the salinity of the wastewater, which was 1.2 % in the activated sludge system of the present study. In common domestic wastewater, as studied by Kwon et al. (2010), the salinity was usually lower than 0.05 %. The activated sludge in this study was a suspended system while in the study of Kwon et al (2010) the activated sludge was an integrated fixed-film system.

Consistent with the high abundance of *Thermotogales* bacteria in the digestion sludge observed in this study, other researchers also reported that there were about 53 % *Thermotogales* bacterium in an anaerobic digester based on cloning results (Sasaki et al. 2010). The *Thermotogales* order contains many different members, which are gram-negative and rod-shaped anaerobic thermopiles containing unique lipids (Huber et al. 1990). The novelty found in the present study was that nearly all of the *Thermotogales* bacteria were affiliated with *Kosmotoga* genus which was a recently proposed new genus (DiPippo et al. 2009) containing members isolated from oil production fluid (*Kosmotoga olearia*) (DiPippo et al. 2009) and from a shallow hydrothermal system occurring within a coral reef (*Kosmotoga arenicorallina*) (Nunoura et al. 2010). So far, this is the first time to report the extremely high abundance (~67 %). As a comparison, only about 0.05 % *Kosmotoga* species was found in another freshwater sludge digester operated under similar conditions. So, it could be very interesting to do further studies to investigate these *Kosmotoga* species in this seawater-containing digester and explore their roles in the sludge digestion.

The results of McLellan et al. (2010) on the bacterial diversity in a WWTP influent showed that *Actinobacteria*, *Bacteroidetes*, and *Firmicutes* were the most dominant three groups of bacteria in the influent, adding up to 37.5 % of the total bacteria. However, in the present study, as shown in Table S2, the top three classes of bacteria were *Deltaproteobacteria*, *Clostridia*, and *Gammaproteobacteria*. At the order level, the top three dominant groups were *Desulfobacterales*, *Clostridiales*, and *Desulfovibrionales*, among which *Desulfobacterales* and *Desulfovibrionales* are the common sulfate-reducing bacteria. The high abundance of sulfate-reducing bacteria that existed in the influent was mainly because of seawater portion in the influent. During transportation of sewage in the pipeline, anaerobic condition led to the blooming of *Desulfobacterales* and *Desulfovibrionales.* Therefore, the dominant bacterial groups in this study were different from those of McLellan et al. (2010).

For the effluent, the present study is the first one which analyzed the whole bacterial diversity symmetrically and comprehensively using 454 pyrosequencing. An important phenomenon found in the present study was the high percentage of *Mycobacterium* and *Vibrio* genera (Fig. 4), which could be potentially pathogenic bacteria and harmful to humans (Bibby et al. 2010). Although it is difficult to tell whether these *Mycobacterium* and *Vibrio* bacteria in the effluent were pathogens since not all species in these genera are pathogenic bacteria (Ye and Zhang, 2011), it deserves further studies to explore the impacts of the bacteria in the effluent. Besides, a few bacterial diversity differences between the activated sludge sample and the effluent sample could be observed from Fig. 4. For example, *Caldilinea*, *Lewinella*, *Leptospira,* etc. were only detected in the activated sample. *Serratia*, *Shewanella*, *Marinomonas*, etc. were found in the effluent sample but not in the activated sample. This is an indication that the sedimentation process may change the abundance of bacteria. Because the bacteria in the activated sludge flocs were removed during the sedimentation process, some free-swimming bacteria populations still remained in the effluent.

To our best knowledge, this is the first study introducing a method to differentiate the “gene percentage” and “cell percentage” by refereeing to the rrnDB (Lee et al. 2009). This approach successfully demonstrated the underestimation or overestimation of some bacterial populations using 16S rRNA gene percentage only. Due to the incomplete data in the rrnDB, the average 16 S rRNA gene copy number was used to represent those data-not-available genera. Such practice may introduce some errors, but these errors could be corrected soon with the rapid development of rrnDB due to the more and more completion of whole genome projects of known bacterial species.

## Electronic supplementary material

Below is the link to the electronic supplementary material.

ESM 1 (DOC 170 kb)
