# Supplementary material for: Bacterial communities in different sections of a municipal wastewater treatment plant revealed by 16S rDNA 454 pyrosequencing
Source: Appl Microbiol Biotechnol. 2012 May 5;97(6):2681–90. doi: 10.1007/s00253-012-4082-4 (PMC3586070; doi:10.1007/s00253-012-4082-4)
Supplement: Supplementary file 1 — (DOC 170 kb) [file 253_2012_4082_MOESM1_ESM.doc]

Electronic Supplementary Material for

Bacterial Communities in Different Sections of a Municipal Wastewater Treatment Plant Revealed by 16S rDNA 454 Pyrosequencing

Lin Ye and Tong Zhang*

Environmental Biotechnology Lab, Department of Civil Engineering, The University of Hong Kong, Hong Kong SAR, China

^*^Corresponding author

Address: Environmental Biotechnology Lab, Department of Civil Engineering, The University of Hong Kong, Pokfulam Road, Hong Kong SAR, China

Email: zhangt@hkucc.hku.hk

Tel: +852-2857 8551

Fax: +852-2859 8987

Table and Figure Captions

**Table S1** Sequences assignment results on phylum level (%)

**Table S2** Sequences assignment results on class level (%)

**Table S3** Sequences assignment results on order level (%)

**Table S4** Environmental data and WWTP operational parameters related to the samples analyzed in this study

**Fig. S1** RDP classifier results showing of high abundance of *Kosmotoga genus* in digestion sludge

**Fig. S2** RDP classifier results of the sequences obtained from cloning

**Fig. S3** Flowchart of Shatin WWTP

**Table S1** Sequences assignment results on phylum level (%)

|  | Activated Sludge | Digestion Sludge | Influent | Effluent |
| --- | --- | --- | --- | --- |
| *Actinobacteria* | 9.33 | 0.22 | 4.74 | 8.10 |
| *Bacteroidetes* | 5.65 | 0.55 | 0.59 | 1.52 |
| *Chlamydiae* | 0.63 | 0.00 | 0.00 | 0.28 |
| *Lentisphaerae* | 0.04 | 0.00 | 0.44 | 0.07 |
| *Verrucomicrobia* | 3.84 | 0.20 | 1.87 | 2.55 |
| *Chloroflexi* | 4.80 | 0.44 | 0.04 | 0.17 |
| *Cyanobacteria* | 0.00 | 0.04 | 0.03 | 0.19 |
| *Deinococcus-Thermus* | 0.22 | 0.00 | 0.04 | 0.18 |
| *Acidobacteria* | 0.60 | 0.00 | 0.07 | 0.13 |
| *Fibrobacteres* | 0.00 | 0.00 | 0.16 | 0.00 |
| *Firmicutes* | 4.49 | 2.83 | 16.49 | 1.19 |
| *Fusobacteria* | 0.00 | 0.00 | 0.31 | 0.00 |
| *Gemmatimonadetes* | 0.10 | 0.03 | 0.00 | 0.03 |
| *Nitrospirae* | 0.69 | 0.00 | 0.00 | 0.05 |
| *Planctomycetes* | 8.45 | 0.07 | 0.19 | 0.97 |
| *Proteobacteria* | 23.82 | 3.82 | 51.39 | 31.24 |
| *Spirochaetes* | 0.81 | 0.15 | 0.33 | 0.03 |
| *Synergistetes* | 0.04 | 4.94 | 2.45 | 0.06 |
| *Tenericutes* | 0.06 | 0.00 | 0.37 | 1.82 |
| *Thermotogae* | 0.16 | 66.96 | 1.59 | 0.15 |
| *Caldithrix* | 0.25 | 0.00 | 0.00 | 0.10 |
| *candidate division OP11* | 0.06 | 0.00 | 0.00 | 1.47 |
| *candidate division OP3* | 0.00 | 0.00 | 0.07 | 0.00 |
| *candidate division SR1* | 0.10 | 0.00 | 0.00 | 0.41 |
| *candidate division TM7* | 1.53 | 0.11 | 0.16 | 1.48 |
| *Ciliophora* | 0.00 | 0.00 | 0.04 | 0.00 |
| *Dinophyceae* | 0.83 | 0.00 | 0.00 | 0.00 |
| *Arthropoda* | 0.00 | 0.00 | 0.00 | 0.04 |
| *Bacillariophyta* | 0.00 | 0.00 | 0.00 | 0.11 |
| *Streptophyta* | 0.05 | 0.00 | 0.00 | 0.00 |

**Table S2** Sequences assignment results on class level (%)

|  | Activated Sludge | Digestion Sludge | Influent | Effluent |
| --- | --- | --- | --- | --- |
| *Actinobacteria (class)* | 9.33 | 0.22 | 4.74 | 8.10 |
| *Bacteroidia* | 0.05 | 0.05 | 0.25 | 0.00 |
| *Cytophagia* | 0.00 | 0.00 | 0.00 | 0.09 |
| *Flavobacteria* | 0.30 | 0.00 | 0.00 | 0.41 |
| *Sphingobacteria* | 2.59 | 0.05 | 0.10 | 0.53 |
| *unclassified Bacteroidetes* | 0.04 | 0.00 | 0.00 | 0.04 |
| *Chlamydiae (class)* | 0.63 | 0.00 | 0.00 | 0.28 |
| *Lentisphaerae* | 0.04 | 0.00 | 0.44 | 0.07 |
| *Opitutae* | 0.00 | 0.00 | 0.00 | 0.06 |
| *Spartobacteria* | 0.00 | 0.00 | 0.00 | 0.04 |
| *unclassified Verrucomicrobia* | 0.23 | 0.03 | 0.00 | 0.98 |
| *Verrucomicrobiae* | 1.89 | 0.00 | 0.00 | 0.89 |
| *Anaerolineae* | 0.37 | 0.14 | 0.00 | 0.04 |
| *Caldilineae* | 4.17 | 0.00 | 0.00 | 0.09 |
| *Dehalococcoidetes* | 0.06 | 0.00 | 0.00 | 0.00 |
| *Thermomicrobia (class)* | 0.03 | 0.00 | 0.00 | 0.00 |
| *unclassified Chloroflexi* | 0.00 | 0.30 | 0.04 | 0.04 |
| *Oscillatoriales* | 0.00 | 0.04 | 0.03 | 0.16 |
| *Deinococci* | 0.22 | 0.00 | 0.04 | 0.18 |
| *Acidobacteria (class)* | 0.60 | 0.00 | 0.00 | 0.07 |
| *Holophagae* | 0.00 | 0.00 | 0.07 | 0.03 |
| *Fibrobacteres (class)* | 0.00 | 0.00 | 0.16 | 0.00 |
| *Bacilli* | 1.78 | 0.87 | 4.37 | 0.29 |
| *Clostridia* | 2.05 | 0.97 | 9.19 | 0.73 |
| *Erysipelotrichi* | 0.08 | 0.00 | 0.39 | 0.03 |
| *Negativicutes* | 0.00 | 0.00 | 1.27 | 0.03 |
| *Fusobacteria (class)* | 0.00 | 0.00 | 0.24 | 0.00 |
| *Gemmatimonadetes (class)* | 0.06 | 0.03 | 0.00 | 0.03 |
| *Nitrospira (class)* | 0.69 | 0.00 | 0.00 | 0.05 |
| *Phycisphaerae* | 0.03 | 0.00 | 0.00 | 0.09 |
| *Planctomycetacia* | 8.24 | 0.07 | 0.16 | 0.85 |
| *Alphaproteobacteria* | 10.41 | 0.16 | 0.77 | 5.95 |
| *Betaproteobacteria* | 2.92 | 0.11 | 0.54 | 2.21 |
| *Deltaproteobacteria* | 2.98 | 0.86 | 39.98 | 1.44 |
| *Epsilonproteobacteria* | 0.00 | 0.00 | 1.15 | 0.05 |
| *Gammaproteobacteria* | 2.08 | 0.37 | 5.20 | 17.76 |
| *Spirochaetes (class)* | 0.81 | 0.15 | 0.33 | 0.03 |
| *Synergistia* | 0.00 | 4.42 | 1.55 | 0.06 |
| *Mollicutes* | 0.06 | 0.00 | 0.37 | 1.82 |
| *Thermotogae (class)* | 0.16 | 66.96 | 1.59 | 0.15 |
| *unclassified Bacteria* | 1.94 | 0.11 | 0.23 | 3.46 |
| *Litostomatea* | 0.00 | 0.00 | 0.04 | 0.00 |
| *Dinophyceae* | 0.83 | 0.00 | 0.00 | 0.00 |
| *Malacostraca* | 0.00 | 0.00 | 0.00 | 0.04 |
| *Bacillariophyta* | 0.00 | 0.00 | 0.00 | 0.11 |
| *Liliopsida* | 0.05 | 0.00 | 0.00 | 0.00 |

**Table S3** Sequences assignment results on order level (%)

|  | Activated Sludge | Digestion Sludge | Influent | Effluent |
| --- | --- | --- | --- | --- |
| *Acidimicrobiales* | 0.24 | 0.00 | 0.00 | 0.00 |
| *Actinomycetales* | 5.13 | 0.10 | 1.04 | 7.20 |
| *Bifidobacteriales* | 0.60 | 0.00 | 3.41 | 0.14 |
| *Coriobacteriales* | 0.00 | 0.00 | 0.15 | 0.00 |
| *Rubrobacterales* | 0.07 | 0.00 | 0.00 | 0.00 |
| *unclassified Actinobacteria* | 0.16 | 0.00 | 0.00 | 0.00 |
| *Bacteroidales* | 0.05 | 0.05 | 0.25 | 0.00 |
| *Cytophagales* | 0.00 | 0.00 | 0.00 | 0.09 |
| *Flavobacteriales* | 0.25 | 0.00 | 0.00 | 0.35 |
| *Sphingobacteriales* | 2.45 | 0.05 | 0.10 | 0.46 |
| *unclassified Bacteroidetes* | 0.04 | 0.00 | 0.00 | 0.04 |
| *Chlamydiales* | 0.63 | 0.00 | 0.00 | 0.28 |
| *Lentisphaerales* | 0.04 | 0.00 | 0.00 | 0.07 |
| *unclassified Lentisphaerae* | 0.00 | 0.00 | 0.07 | 0.00 |
| *Victivallales* | 0.00 | 0.00 | 0.17 | 0.00 |
| *Puniceicoccales* | 0.00 | 0.00 | 0.00 | 0.06 |
| *Spartobacteria* | 0.00 | 0.00 | 0.00 | 0.04 |
| *unclassified Verrucomicrobia* | 0.23 | 0.03 | 0.00 | 0.98 |
| *Verrucomicrobiales* | 1.89 | 0.00 | 0.00 | 0.89 |
| *Anaerolineales* | 0.37 | 0.14 | 0.00 | 0.04 |
| *Caldilineales* | 4.17 | 0.00 | 0.00 | 0.09 |
| *Dehalococcoidetes* | 0.06 | 0.00 | 0.00 | 0.00 |
| *Sphaerobacterales* | 0.03 | 0.00 | 0.00 | 0.00 |
| *unclassified Chloroflexi* | 0.00 | 0.30 | 0.04 | 0.04 |
| *Oscillatoriales* | 0.00 | 0.04 | 0.03 | 0.16 |
| *Deinococcales* | 0.22 | 0.00 | 0.04 | 0.18 |
| *Acidobacteriales* | 0.60 | 0.00 | 0.00 | 0.07 |
| *Holophagales* | 0.00 | 0.00 | 0.07 | 0.03 |
| *Fibrobacterales* | 0.00 | 0.00 | 0.16 | 0.00 |
| *Bacillales* | 0.45 | 0.00 | 0.00 | 0.12 |
| *Lactobacillales* | 1.32 | 0.87 | 4.33 | 0.17 |
| *Clostridiales* | 1.95 | 0.51 | 8.24 | 0.68 |
| *Erysipelotrichales* | 0.08 | 0.00 | 0.39 | 0.03 |
| *Selenomonadales* | 0.00 | 0.00 | 1.27 | 0.03 |
| *Fusobacteriales* | 0.00 | 0.00 | 0.24 | 0.00 |
| *Gemmatimonadales* | 0.06 | 0.03 | 0.00 | 0.03 |
| *Nitrospirales* | 0.69 | 0.00 | 0.00 | 0.05 |
| *Phycisphaerales* | 0.03 | 0.00 | 0.00 | 0.09 |
| *Planctomycetales* | 8.12 | 0.07 | 0.16 | 0.85 |
| *unclassified Planctomycetacia* | 0.07 | 0.00 | 0.00 | 0.00 |
| *Caulobacterales* | 0.00 | 0.00 | 0.00 | 0.03 |
| *Parvularculales* | 0.00 | 0.03 | 0.00 | 0.03 |
| *Rhizobiales* | 4.86 | 0.05 | 0.22 | 0.56 |
| *Rhodobacterales* | 1.05 | 0.05 | 0.23 | 0.23 |
| *Rhodospirillales* | 0.10 | 0.00 | 0.00 | 0.04 |
| *Rickettsiales* | 0.00 | 0.00 | 0.00 | 0.05 |
| *Sphingomonadales* | 0.03 | 0.00 | 0.00 | 0.09 |
| *unclassified Alphaproteobacteria* | 0.30 | 0.00 | 0.00 | 0.12 |
| *Burkholderiales* | 1.19 | 0.05 | 0.11 | 0.24 |
| *Hydrogenophilales* | 0.05 | 0.00 | 0.00 | 0.00 |
| *Methylophilales* | 0.00 | 0.00 | 0.00 | 0.70 |
| *Neisseriales* | 0.00 | 0.00 | 0.05 | 0.00 |
| *Nitrosomonadales* | 0.14 | 0.00 | 0.00 | 0.07 |
| *Rhodocyclales* | 1.07 | 0.05 | 0.18 | 0.15 |
| *Desulfobacterales* | 0.05 | 0.06 | 22.25 | 0.42 |
| *Desulfovibrionales* | 0.06 | 0.41 | 4.56 | 0.13 |
| *Desulfuromonadales* | 0.07 | 0.00 | 0.21 | 0.00 |
| *Myxococcales* | 1.21 | 0.00 | 0.00 | 0.00 |
| *Syntrophobacterales* | 0.19 | 0.07 | 0.00 | 0.05 |
| *unclassified Deltaproteobacteria* | 0.00 | 0.00 | 0.00 | 0.03 |
| *Campylobacterales* | 0.00 | 0.00 | 0.06 | 0.00 |
| *Alteromonadales* | 0.00 | 0.00 | 1.60 | 0.32 |
| *Chromatiales* | 0.03 | 0.00 | 0.03 | 0.14 |
| *Enterobacteriales* | 0.00 | 0.00 | 0.00 | 0.15 |
| *Legionellales* | 0.06 | 0.00 | 0.05 | 0.63 |
| *Oceanospirillales* | 0.00 | 0.00 | 0.09 | 0.22 |
| *Pasteurellales* | 0.04 | 0.00 | 0.05 | 0.00 |
| *Pseudomonadales* | 0.18 | 0.00 | 0.38 | 4.02 |
| *Thiotrichales* | 0.03 | 0.00 | 1.54 | 0.06 |
| *unclassified Gammaproteobacteria* | 0.66 | 0.12 | 0.00 | 0.11 |
| *Vibrionales* | 0.00 | 0.00 | 0.43 | 4.27 |
| *Xanthomonadales* | 0.35 | 0.07 | 0.13 | 0.85 |
| *Spirochaetales* | 0.78 | 0.15 | 0.33 | 0.03 |
| *Synergistales* | 0.00 | 4.42 | 1.55 | 0.06 |
| *Acholeplasmatales* | 0.03 | 0.00 | 0.37 | 1.01 |
| *Entomoplasmatales* | 0.03 | 0.00 | 0.00 | 0.19 |
| *Mycoplasmatales* | 0.00 | 0.00 | 0.00 | 0.54 |
| *Thermotogales* | 0.16 | 66.96 | 1.59 | 0.15 |
| *unclassified Bacteria* | 1.94 | 0.11 | 0.23 | 3.46 |
| *Vestibuliferida* | 0.00 | 0.00 | 0.04 | 0.00 |
| *Gymnodiniales* | 0.83 | 0.00 | 0.00 | 0.00 |
| *Decapoda* | 0.00 | 0.00 | 0.00 | 0.04 |
| *Bacillariophyta* | 0.00 | 0.00 | 0.00 | 0.11 |
| *Alismatales* | 0.05 | 0.00 | 0.00 | 0.00 |

**Table S4** Environmental data and WWTP operational parameters related to the samples analyzed in this study

| **Sample** | **Environmental data and WWTP operational parameters** |
| --- | --- |
| Influent | COD: 358 mg/l  BOD_5_: 173 mg/L  Total Suspended Solid (TSS): 370 mg/L  Total Nitrogen: 33 mg/L  Ammonium Nitrogen: 20 mg/L  Total Phosphorus 3.7 mg/L  pH: 7.2 |
| Effluent | COD: 49 mg/l  BOD_5_: 5.2 mg/L  TSS: 6.7 mg/L  Total Nitrogen: 6.5 mg/L  Ammonium Nitrogen: 1.1 mg/L  Total Phosphorus 1.7 mg/L  pH: 7.6 |
| Aeration Tank and  Activated Sludge | Activated sludge system, no fixed materials (carriers) for bacteria (biofilm) growth  DO: ~2.0 mg/L  Temperature: ~25℃  pH: 7.5  Hydraulic Retention Time (HRT): 9 h  Solid Retention Time (SRT): 12 d  Loading: ~0.4 kg COD/(kg VSS.d)  Specific sludge yield: 0.5 kg VSS/(kg BOD_5_·d) |
| Digester and  Digestion Sludge | HRT: 13.3 d  pH: 6.7  H_2_S concentration: 143 mg/L  Methane in biogas: 60%  Volatile Solid (VS): 46% of TS  Total Solid (TS): 3.7%  Temperature: 37℃ |


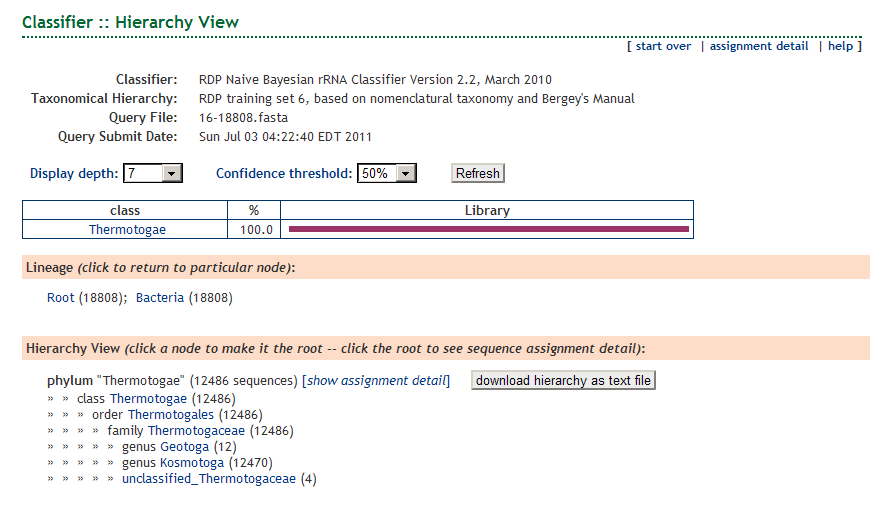


**Fig. S1** RDP classifier results showing high abundance of *Kosmotoga* genus in digestion sludge


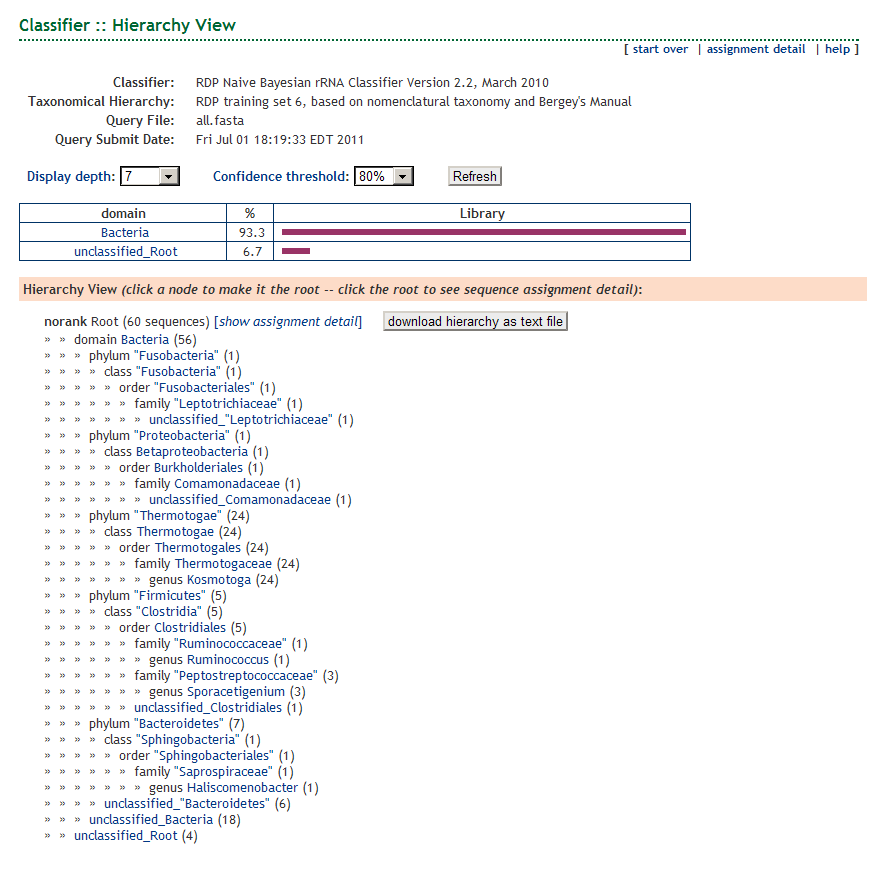


**Fig. S2** RDP classifier results of the sequences obtained from cloning

A

Sewage

Effluent

Grit Channel

Primary Sedimentation Tank

Aeration Tank

Final Sedimentation Tank

Air Floatation Unit

Returned Activated Sludge

Digestor

Dewatering Unit

Dewatered Sludge to Landfill Site

Thickened Activated Sludge

Primary Sludge

B

C

D

**Fig. S3** Flowchart of Shatin WWTP. The Influent, Activated Sludge, Effluent and Digestion Sludge samples were take from Site A, B, C and D, respectively.
